# Long-term psychological consequences of forced displacement: identity and belonging in Egypt’s resettled Nubian community

**DOI:** 10.3389/fpsyg.2026.1852510

**Published:** 2026-09-23

**Authors:** Shimaa Abdelaziz, Xiao Han, Edris Alam, Guoqing Shi

**Affiliations:** 1 School of Public Administration, Hohai University, Nanjing, China; 2 School of Social Sciences, Monash University, Clayton, VIC, Australia; 3 Faculty of Resilience, Rabdan Academy, Abu Dhabi, United Arab Emirates; 4 Department of Geography and Environmental Studies, University of Chittagong, Chittagong, Bangladesh

**Keywords:** alienation, cultural identity, forced displacement, nostalgia, Nubians, psychosocial support, social identity theory

## Abstract

This study investigates the long-term psychological and identity consequences of forced displacement on the Nubian community resettled in Kom Ombo, Aswan, over six decades after the construction of the Aswan High Dam, one of the most significant cases of development-induced displacement in the Arab world. The study addresses two research questions: what are the psychological impacts of forced displacement on the sense of belonging among the Nubian community resettled in Kom Ombo, and what psychological and identity patterns does forced displacement reveal among the Nubian community, and how does cultural identity function as a mediator and moderator in the relationship between alienation and belonging. A mixed-methods approach was adopted, combining a quantitative survey (*n* = 382) with in-depth interviews (*n* = 30) and key informant interviews (*n* = 30). Statistical analyses included exploratory factor analysis, cluster analysis, MANOVA, and mediation and moderation analyses, complemented by thematic analysis of qualitative data. Findings revealed four psychological dimensions of displacement-related distress and four distinct adaptation profiles. Persistent alienation was reported by 34.6%, and loss of inner security by 44.2%, while 47.9% reported partial identity loss, and 48.2% actively preserved the Nubian language. MANOVA identified education level (Wilks’ Λ = 0.897, *p* = 0.001) and marital status (Wilks’ Λ = 0.919, *p* = 0.001) as the only significant demographic predictors. A counterintuitive positive association between alienation and belonging was identified: higher alienation from mainstream society was associated with stronger attachment to the Nubian community (β = 0.250, *p* < 0.001). A significant indirect association between Cultural Identity and Belonging through Alienation was identified, with Alienation accounting for 26.5% of the statistical association (Sobel Z = 3.24, *p* = 0.001). Cultural Identity also significantly moderated the Alienation–Belonging relationship (β = −0.143, *p* = 0.028). Qualitative findings further illustrated that nostalgia, language preservation, and communal practices function as active strategies for cultural resistance and identity maintenance. The study concludes that post-displacement Nubian identity is dynamically negotiated between loss and resistance, and that cultural strength constitutes a genuine psychosocial resource. Differentiated interventions informed by psychological profiles and grounded in cultural recognition are recommended.

## 1 Introduction

Development-induced displacement and resettlement are widespread outcomes of large-scale projects, particularly dam construction, affecting displaced populations and host communities alike (Tang et al., 2022). Although displacement is frequently associated with adverse consequences, post-relocation conditions may also include socioeconomic improvements, depending on the context and implementation of resettlement programs, while livelihood, social, and adjustment challenges may persist (Kirchherr et al., 2016; Tao and Shi, 2023). Recent scholarship, therefore, conceptualizes displacement as a multidimensional process extending beyond physical relocation to encompass interconnected economic, social, psychological, and cultural dimensions (Bose, 2018). While resettlement policies often prioritize material compensation and physical reconstruction, less visible impacts such as social fragmentation, emotional distress, and psychological wellbeing may receive comparatively less attention (Le Hang et al., 2022). Evidence from dam-induced resettlement highlights a persistent erosion of social status, entitlements, and community cohesion, alongside the spatial exclusion and reduced participation in communal life, which collectively increase social isolation and reduce empowerment (Tilt and Gerkey, 2016). Displacement also weakens social capital and kinship-based networks, undermining adaptive capacity and deepening vulnerability, particularly among marginalized groups (Nhung and Thang, 2017). Psychologically, resettlement has been described as a totalizing experience associated with grief, anxiety, insecurity, and longer-term distress, with impacts that may persist across generations (Rieber et al., 2025). Social connectedness and belonging can buffer these pressures, whereas stigmatization and spatial marginalization exacerbate alienation and weaken attachment to place (Song et al., 2024).

In response to rupture and loss, displaced groups often rely on nostalgia, storytelling, and memory work to restore continuity and reconstruct identity (Xi and Hwang, 2011). However, nostalgia can be ambivalent, providing comfort while also intensifying alienation and complicating integration when new environments are perceived as culturally incongruent (Nakayama and Fujikura, 2019). These dynamics are especially salient in the Nubian displacement following the Aswan High Dam, which produced enduring cultural and psychological dislocation for resettled communities, including those in Kom Ombo (Gilmore, 2016). Nubian nostalgia for “Old Nubia” has been documented as both coping and cultural resistance, expressed through music, oral traditions, and displacement songs that preserve communal memory across generations(Agha, 2019; Kaddal, 2021). Against this background, our study aimed to understand the following research question:

1. What are the psychological impacts of forced displacement on the sense of belonging among the Nubian community resettled in Kom Ombo?
2. What psychological and identity patterns does forced displacement reveal among the Nubian community, and how does cultural identity function as a mediator and moderator in the relationship between alienation and belonging?

## 2 Literature review

### 2.1 Development-induced displacement and resettlement impact on wellbeing

Foundational scholarship on development-induced displacement and resettlement (DIDR) conceptualizes resettlement as a multidimensional process extending beyond physical relocation. Cernea’s Impoverishment Risks and Reconstruction (IRR) model identifies interconnected risks such as landlessness, marginalization, and social disarticulation, while also emphasizing the possibility of reconstruction through the restoration of livelihoods and community structures (Cernea, 1997, 2000a). Scudder (2005) similarly conceptualizes resettlement as a long-term process involving stress, adaptation, and potential reconstruction. This literature indicates that post-resettlement outcomes cannot be reduced to economic loss or assumed to be uniformly negative; rather, they vary according to the social, institutional, and cultural conditions surrounding relocation and reconstruction. At the level of wellbeing, these processes operate through interrelated economic, social, and psychosocial pathways. Even when resettlement improves housing conditions and expands access to social insurance, evidence indicates persistent livelihood disruption, reflected in reduced employment, lower income, and weakened livelihood resources, suggesting that material gains do not necessarily translate into livelihood recovery (Huang et al., 2018). These impacts may be amplified among marginalized groups when displacement disrupts cultural identity and traditional livelihoods, while governance failures, such as corruption and politicization, delay compensation and intensify vulnerability (Priyadarshini and Dulla, 2023). Beyond economic effects, displaced populations frequently report psychosocial decline associated with community loss, inadequate infrastructure, insecurity, and increased morbidity, with heightened risks documented among youth (Nhung and Thang, 2017; Nikuze et al., 2019; Opoku et al., 2024). Wellbeing is further shaped by social inclusion, as opportunities for belonging and integration within host settings are particularly consequential for youth and vulnerable groups (Correa-Velez et al., 2010). In this context, the disruption of social capital and community networks reduces social support, weakens resilience, and constrains adaptive capacity following resettlement (Xu et al., 2022).

### 2.2 Identity disruption and reconstruction after relocation

Relocation commonly disrupts identity by separating individuals from familiar places, everyday practices, and community-based meaning-making, making identity reconstruction a central dimension of post-relocation adaptation (Lejano et al., 2018). In dam-affected contexts, resettlement is linked to identity crises, cultural dislocation, and emotional trauma, showing that adjustment after relocation goes beyond material recovery to rebuilding place- and culture-based self-understandings. Across settings, forced relocation can fracture identity yet also spur reconstruction. For example, Syrian refugee families reportedly rebuild collective identity through reorganized family collaboration, strategic consumption, and community engagement, supporting resilience and reshaping roles (Alhanouti, 2025). Comparable patterns appear among Tibetan refugees, where identity trajectories differ by place of birth, yet both Tibetan-born and Indian-born individuals actively reconstruct identity by integrating cultural elements through productization (Ramanathan and Singh, 2025). Place-based narratives further illustrate that identity disruption is relational and strongly tied to loss of a familiar city or the gradual formation of attachment to a new one, highlighting the enduring influence of place on identity formation after relocation. Consistent with this, relocation has been linked to reduced wellbeing, increased anxiety, diminished community-based identity, and lower perceived safety, particularly when social cohesion and cultural ties are not effectively rebuilt (Yang et al., 2024). Accordingly, effective recovery strategies increasingly emphasize that long-term adaptation requires attention to emotional, social, and cultural dimensions of identity, including resident participation in decision-making, support for rebuilding social networks, and recognition of cultural identities (Kamila et al., 2026).

### 2.3 Social cohesion, belonging, and community continuity in resettled groups

Social cohesion and belonging in resettlement contexts can be understood in part through a social capital perspective, which emphasizes the resources embedded in social relationships and networks that support trust, reciprocity, and collective continuity (Portes, 1998). Previous studies indicate that social cohesion and a sense of belonging among resettled groups are often weakened by negative social connotations, stigmatization, and low self-esteem, which can undermine attachment to place and reduce participation in community life (Lakshman and Azam, 2023). In this context, maintaining close-knit social groups during relocation has been emphasized as an important condition for sustaining community continuity and strengthening belonging in resettlement settings (Lakshman and Azam, 2023). Evidence from internal displacement further shows that relocation processes can erode trust, disrupt community continuity, and weaken social networks, with implications for both displaced groups and host communities. For example, research in Benue State reports that displacement undermines cohesion and trust, suggesting that integration programs and equitable resource distribution are central for rebuilding social networks and fostering belonging between internally displaced persons and host communities (Zamani et al., 2025). Similar conclusions emerge in host-community research from Ukraine, which highlights that strengthening social cohesion requires a comprehensive approach addressing socio-economic, socio-psychological, and cultural-communicative challenges affecting displaced persons and host settings (Chuiko and Ilchuk, 2024). These social-relational processes provide part of the broader context within which the present study employs Social Identity Theory as a theoretical lens for understanding how belonging, recognition, and identity threat shape post-resettlement experiences.

Previous research indicates that displacement and resettlement involve interconnected processes of psychosocial disruption, identity reconstruction, and changing social relationships. However, these dimensions are often examined separately, leaving a limited understanding of how alienation, cultural identity, social support loss, and psychological belonging are interrelated within long-resettled communities. The Nubian case provides an opportunity to examine these relationships within a population whose resettlement experience extends across generations. This gap provides the basis for the research questions and hypotheses developed in the present study.

## 3 Theoretical framework

Social Identity Theory (SIT) explains how individuals derive part of their self-concept from membership in social groups, shaping attitudes, emotions, and behavior through processes of identification and belonging (Hogg, 2016). Developed by Tajfel and Turner, SIT distinguishes between personal identity and social identity, emphasizing that group-based identification includes cognitive recognition of membership, emotional attachment, and evaluative judgment of the group’s value (Tajfel and Turner, 2004). Because group membership often becomes especially salient under conditions of social change, displacement, or perceived threat, SIT has been widely applied to explain intergroup relations and the ways individuals respond to shifting social boundaries (Cadsby et al., 2016).

SIT proposes that social identity forms through three core processes. First, individuals categorize themselves and others into social groups, creating perceived in-groups and out-groups (Bouncken et al., 2022). Second, they engage in social comparison, evaluating the in-group relative to out-groups to achieve positive distinctiveness and protect self-esteem (Kanzola et al., 2024). Third, identification develops as a sense of belongingness that increases alignment with group norms and shared meanings, shaping attitudes and behaviors in ways that maintain group coherence (Chen et al., 2024). Under strong group salience, depersonalization may occur, meaning that perceptions and responses are guided more by the group prototype than by purely individual preferences (Abrams, 2001). Importantly, contemporary work treats SIT as dynamic, with identity continuously shaped through interaction between individuals and social contexts rather than as a fixed attribute (Curşeu et al., 2020).

Within the broader context of development-induced displacement, Social Identity Theory (SIT) remains the primary psychological framework of the present study, while complementary perspectives on place attachment, social capital, and post-resettlement adaptation help situate identity and belonging within their broader social and spatial context. Place attachment highlights the emotional and symbolic significance of meaningful environments (Scannell and Gifford, 2010), whereas social capital draws attention to the resources embedded in social relationships and networks that can support collective life and community continuity (Portes, 1998). In displacement settings, the disruption and reconstruction of these social and place-based resources form part of the longer-term processes of stress, adjustment, and reconstruction described in the DIDR literature (Cernea, 2000b; Scudder, 2005). These perspectives are therefore used as complementary interpretive lenses rather than as separate competing theories.

Within this theoretical framework, SIT offers a basis for anticipating the relationship between alienation and belonging examined in this study: under conditions of perceived exclusion, group boundaries may become more salient, increasing attachment to the cultural in-group rather than weakening it (Tajfel and Turner, 2004; Hogg, 2016). Cultural identity is relevant in two distinct ways: a salient cultural identity may heighten sensitivity to perceived exclusion while reinforcing in-group attachment, and the degree to which identity is already well-established may shape the strength of the association between alienation and compensatory in-group belonging. Alienation, cultural identity, and belonging are thus understood as interrelated processes operating through more than one theoretical pathway.

Against this broader theoretical background, SIT is used to interpret post-resettlement experiences within the Nubian community through belonging, recognition, and identity threat. Alienation and reduced inner security indicate weakened identity continuity and recognition in the new setting. Variation in social isolation reflects fluctuating identification, intensifying when in-group cohesion and social participation decline. Conditional acceptance is interpreted as boundary-making that reinforces in-group and out-group distinctions. Perceived partial identity loss alongside active language preservation is treated as identity-protective behavior under threat, aimed at sustaining collective meaning and self-esteem when heritage is devalued.

## 4 Methodology

To understand the psychological impact and identity formation associated with displacement among the Nubian community in Kom Ombo, Aswan Governorate, this study applied a mixed-methods approach (Tashakkori and Teddlie, 2010), collecting data through questionnaire surveys (quantitatively), key informant interviews (KIIs), and in-depth interviews (IDIs) (qualitatively). Each has its limitations (Gregory et al., 2011). However, mixed methods provide more robust results than relying on solely qualitative or quantitative approaches (Greene et al., 1989; Henn et al., 2006).

### 4.1 Study area

This section outlines the geographical, historical, cultural, and socio-economic context of Wadi Kom Ombo and its relevance to the study of long-term Nubian displacement and resettlement.

Prior to displacement, Egyptian Nubia extended for approximately 320 km along the Nile south of Aswan and included culturally and linguistically differentiated communities, principally the Kenuz in the north, Arab Nubian groups in the central area, and the Fadija in the south (Fahim, 1981). Approximately 48,000 Egyptian Nubians were resettled between 1963 and 1964 in connection with the construction of the Aswan High Dam (Scudder, 2016). The affected communities were transferred to purpose-built settlements primarily around Kom Ombo, north of Aswan, in a setting spatially separated from the Nile. Although migration to urban centers had begun before the High Dam, the inundation necessitated the relocation of the remaining communities in the affected Egyptian Nile Valley. The new setting differed sharply from the riverine environment that had historically shaped Nubian livelihoods, settlement patterns, and cultural life (Fahim, 2010).

The displacement area of Wadi Kom Ombo is located approximately 12 km east of the Nile and is bordered by the Red Sea Mountains to the north, east, and south, and by Kom Ombo to the west. It extends for approximately 60 km in length and about 3 km in width (Figure 1). This location contrasted sharply with the riverine setting to which Nubian communities had historically been accustomed. The relatively limited area allocated for village construction resulted in a more compact settlement pattern (Mohamed, 2018). The Nubian community now occupies a compact area in Kom Ombo, North Aswan, where housing and essential amenities, including schools and health facilities, have been established.

**Figure 1:**
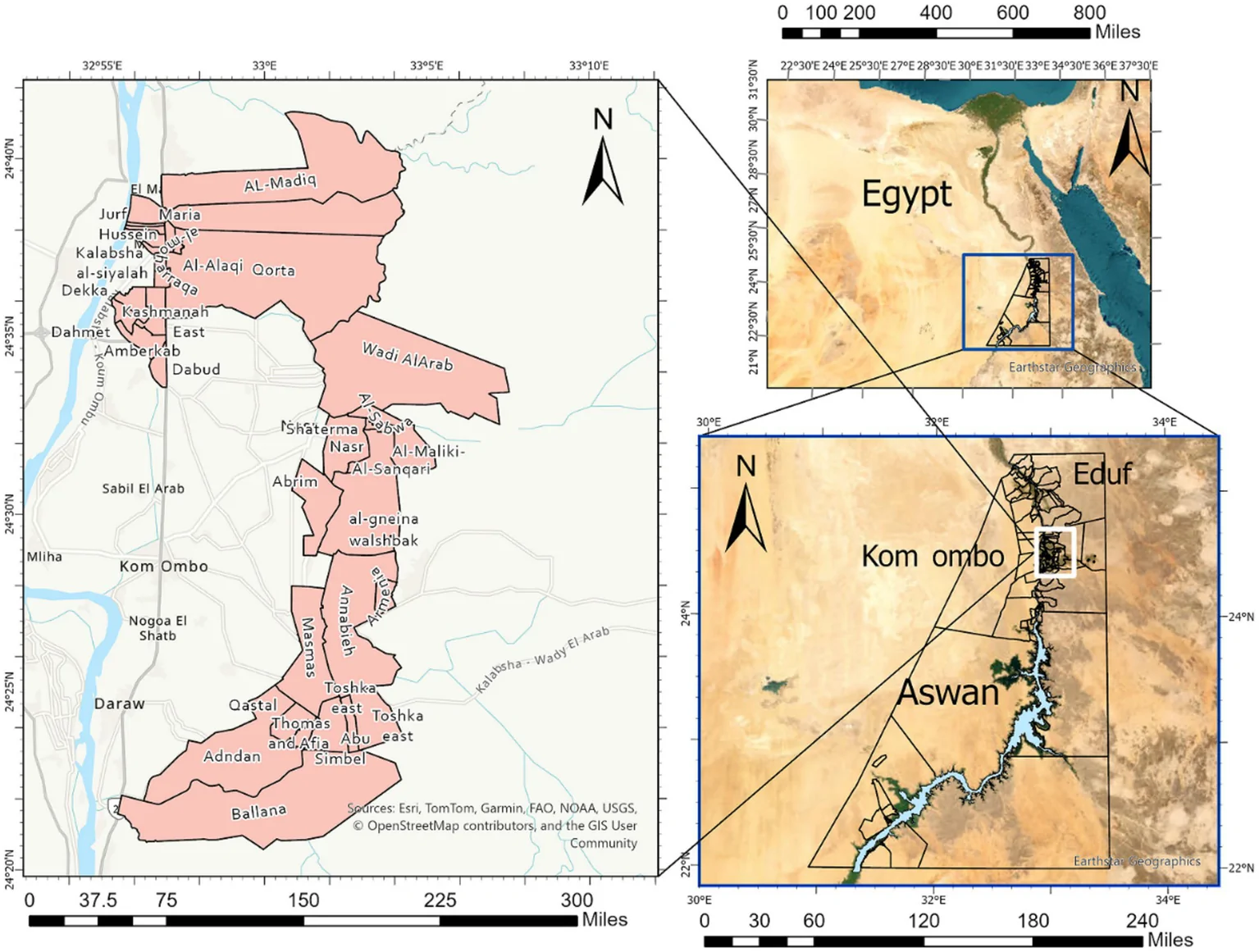
Study area new Nubian.

The receiving environment was a purpose-built resettlement area rather than a conventional host-community setting (Janmyr, 2017). Housing, schools, health facilities, and other infrastructure were developed to accommodate the relocated population, while the spatial organization of the settlement differed substantially from that of Old Nubia (Allam, 2013). Importantly, the resettlement experience was not uniformly negative. Scudder (2016) identifies the Egyptian Nubian case as one of the relatively few large dam-induced resettlement cases in which the living standards of the majority improved over the long term. However, improvements in material living conditions could coexist with the loss of cultural meanings associated with the Nile, ancestral settlements, and established patterns of community life. These characteristics of the resettlement environment provide important context for interpreting experiences of alienation, belonging, and identity continuity examined in the present study. The Nubian case was selected because the community’s long-term experience of development-induced displacement provides a particularly relevant context for examining cultural continuity, identity reconstruction, and psychological adaptation across generations.

### 4.2 Data sources and instruments

*Primary data*: A structured questionnaire employing a three-point response format (Never/Sometimes/Always) was administered in a face-to-face manner to a sample of *n* = 382 adults. Key Informant Interviews (KIIs) were conducted with a diverse group of academics, local leaders/elders, and representatives from non-governmental organizations (*n* = 30). In-Depth Interviews (IDIs) were carried out with community members, ensuring representation across various age and gender demographics (*n* = 30).

The social-psychological component of the questionnaire comprised 20 items organized into four composite scales: Alienation and Grief, Cultural Identity, Social Support Loss, and Psychological Belonging. The Alienation and Grief scale comprised six items assessing alienation, social isolation, loss of inner security, sadness associated with displacement, and psychological discomfort. The Cultural Identity scale comprised five items assessing perceived identity acceptance, identity-related tension, perceived identity loss, language preservation, and emotional responses to heritage neglect. The Social Support Loss scale comprised five items assessing perceived erosion of community support, family ties, family warmth, anxiety associated with weakened social ties, and the perceived quality of relationships in the new environment. The Psychological Belonging scale comprised four items assessing belonging and non-belonging in the current social environment, desire to return to the ancestral homeland, persistent lack of attachment to the resettlement setting, and identity recognition. The study operationalizes these four scales as the principal composite constructs in the quantitative component. Related concepts, including nostalgia, resilience, adaptation, and cultural resistance, are used within the broader interpretation of specific survey patterns and qualitative themes.

*Secondary data sources* included government and archival records, peer-reviewed scholarly articles, books, and reports from non-governmental organizations that pertain to the themes of Nubian displacement and subsequent resettlement.

### 4.3 Data collection procedures

Survey data were obtained through the administration of surveys within the domestic settings of the New Nubian communities located in Wadi Kom Ombo. A stratified, household-based methodology was employed across the various villages, utilizing a systematic selection process derived from locally compiled household registries. Proportional allocation was implemented to distribute the sample appropriately across the villages, in accordance with their demographic composition.

Qualitative data were collected through key informant interviews and comprehensive interviews. Key informants were intentionally selected to represent a range of institutional and historical viewpoints, encompassing community leaders, local officials, and service providers.

### 4.4 Data management and analysis

Quantitative data were analyzed in Python using specialized libraries for multivariate statistics, factor analysis, clustering, and regression-based mediation and moderation analyses, providing a transparent and reproducible analytical framework. Given the cross-sectional nature of the quantitative component, relationships among the study variables were interpreted as statistical associations rather than evidence of causal effects or temporal ordering.

Qualitative data derived from the key informant interviews and in-depth interviews were audio recorded with the requisite consent, transcribed verbatim, and subsequently de-identified prior to analysis. The transcripts were imported into NVivo for systematic organization and coding. The analysis adhered to a thematic approach, involving iterative familiarization with the transcripts, initial open coding, consolidation of codes into a comprehensive codebook, and refinement into higher-order themes that corresponded with the study’s objectives. Patterns were interpreted by contrasting themes across participants and interview types to facilitate an integrated understanding of psychological impacts and the formation of identity.

## 5 Results

The results are organized into three main sections: (1) demographic characteristics and sample representativeness, (2) psychological impacts of displacement encompassing alienation, social isolation, nostalgia, and their underlying factor structure, and (3) identity formation processes including emotional responses, conditional acceptance, and language preservation patterns. Throughout the presentation, quantitative findings are complemented by qualitative insights from key informant interviews (KIIs) and in-depth interviews (IDIs) to provide a nuanced interpretation.

### 5.1 Sample characteristics and representativeness

The 382 survey respondents demonstrated several distinctive demographic patterns that contextualize the subsequent findings (Table 1). The sample was predominantly middle-aged, with 42.1% (*n* = 161) aged 31–45 years and 27.5% (*n* = 105) aged 46–60 years. Given that the major relocation occurred in 1963–1964, respondents in these age groups were born after the displacement and therefore did not experience the relocation directly. Only 8.9% (*n* = 34) were over 61 years, representing the oldest generation in the sample, among whom direct memories of the displacement period may have been possible depending on their age at the time of relocation. Accordingly, the experiences reported by most respondents are interpreted in relation to post-resettlement lived experience, family narratives, intergenerational transmission, and collective memory rather than direct displacement experience alone. The gender distribution revealed a female majority (57.6%, *n* = 220), which may reflect higher female availability for survey participation, greater willingness to discuss family and community issues, or male outmigration for employment.

**Table 1:** Demographic characteristics of survey respondents (*N* = 382).

Variable	Category	*n*	%
Age	18–30 years	82	21.5
	31–45 years	161	42.1
	46–60 years	105	27.5
	More than 61 years	34	8.9
Gender	Female	220	57.6
	Male	162	42.4
Education Level	Illiterate	12	3.1
	Intermediate education	91	23.8
	Above intermediate education	40	10.5
	University graduate	197	51.6
	Post-studies	42	11.0
Marital Status	Single	98	25.7
	Married	242	63.4
	Divorced	21	5.5
	Widowed	21	5.5
Family Size	1–3 members	111	29.1
	4–6 members	225	58.9
	7–10 members	35	9.2
	More than 10 members	11	2.9
Occupation	Government employee	135	35.3
	Private sector employee	106	27.7
	Self-employed	66	17.3
	Agricultural sector	5	1.3
	Others	70	18.3

Perhaps most striking is the exceptionally high educational attainment, with 51.6% holding university degrees and an additional 11.0% possessing postgraduate qualifications. Combined, 62.6% of the sample had completed higher education substantially above typical rates in rural Egyptian populations. This educational profile may reflect either successful post-displacement educational initiatives or sampling bias toward more educated individuals who were more accessible or willing to participate. The occupational distribution revealed the most dramatic transformation: only 1.3% (*n* = 5) currently work in agriculture, representing a near-complete abandonment of traditional Nubian farming livelihoods. Instead, respondents predominantly worked in government positions (35.3%) or the private sector (27.7%), indicating fundamental economic restructuring following displacement. Family size showed a transition toward nuclear arrangements, with 58.9% reporting 4–6 family members, contrasting with the extended family structures that characterized traditional Nubian villages.

### 5.2 Psychological dimensions of displacement: factor structure and internal consistency

Before examining specific psychological experiences associated with displacement, exploratory factor analysis (EFA) was conducted to identify the underlying dimensional structure of the 20 psychological wellbeing items. The analysis revealed four distinct latent factors that collectively explained the variance in displacement-related distress. Factor 1, labeled “Grief and Emotional Distress,” was defined primarily by sadness about pre-displacement life (λ = −0.994) and belief that psychological comfort may never return (λ = −0.993). Factor 2, “Displacement-Related Alienation,” loaded most heavily on feelings of alienation since leaving Nubia (λ = −0.975), increased sadness from village memories (λ = −0.974), and loss of inner security (λ = −0.965). Factor 3, “Cultural Heritage Concern,” encompassed anger when heritage is neglected (λ = −0.778) and desire for cultural understanding (λ = −0.674). Factor 4, “Cultural Adaptation and Isolation,” combined language preservation efforts (λ = −0.561) with feelings of social isolation (λ = 0.506), suggesting tension between maintaining cultural distinctiveness and achieving social integration.

This four-factor structure validates the theoretical distinctions between grief, alienation, identity concerns, and belonging as separate but related psychological constructs. It also aligns with Social Identity Theory’s emphasis on multifaceted identity processes under threat. Table 2 presents the factor loadings for all survey items across the four extracted factors.

**Table 2:** Factor loadings—4-factor exploratory factor analysis (EFA) solution.

Survey item	F1: Grief	F2: Alienation	F3: Heritage	F4: Adaptation
I feel sad remembering life before displacement	−0.994	−0.105	–	–
My psychological comfort may never return	−0.993	-	–	–
I feel alienated since leaving Nubia	0.216	−0.975	–	–
Memories of Nubian village increase my sadness	0.219	−0.974	–	–
I have lost my inner security since displacement	0.205	−0.965	–	–
I get angry when others neglect our heritage	–	–	−0.778	–
I wish someone could understand being Nubian	–	–	−0.674	–
I try to preserve the Nubian language	–	–	–	−0.561
I constantly feel isolated in the new society	–	0.137	–	0.506

Only loadings ≥ |0.30| shown; − = below threshold. F1 = Grief & Emotional Distress; F2 = Displacement-Related Alienation; F3 = Cultural Heritage Concern; F4 = Cultural Adaptation and Isolation.

### 5.3 Psychological experiences associated with displacement

#### 5.3.1 Alienation, security loss, and bifurcated adaptation

Table 3 presents the distribution of responses across six items measuring alienation, isolation, and sadness. The findings reveal a substantial bifurcation in how respondents experienced psychological displacement. Regarding alienation from their original villages, 34.6% reported persistent feelings (responding “Always”), while 44.2% never experienced such alienation, and 21.2% experienced it intermittently. This polarization suggests that while some community members remained psychologically anchored to their place of origin, others successfully detached or adapted to new environments. The loss of inner security showed an even more pronounced pattern, with 44.2% consistently reporting this loss (*M* = 2.10, SD = 0.882), 34.3% never experiencing it, and 21.5% fluctuating. The high percentage reporting constant security loss indicates that, for nearly half of the sample, displacement disrupted not just physical location but also fundamental psychological stability and ontological security.

**Table 3:** Psychological distress indicators among displaced Nubians (*N* = 382).

Indicator	Never	Sometimes	Always	M	SD
	*f* (%)	*f* (%)	*f* (%)		
Alienation since leaving village	169 (44.2)	81 (21.2)	132 (34.6)	2.10	0.88
Constant isolation in new society	152 (39.8)	175 (45.8)	55 (14.4)	1.75	0.69
Lost inner security	131 (34.3)	82 (21.5)	169 (44.2)	2.10	0.88
Sadness remembering past life	137 (35.9)	178 (46.6)	67 (17.5)	2.11	0.90
Memories increase sadness	132 (34.6)	169 (44.2)	81 (21.2)	2.10	0.88
Psychological comfort may never return	136 (35.6)	68 (17.8)	178 (46.6)	2.11	0.90

In contrast, social isolation appeared less severe and more situational. Only 14.4% reported constant isolation in the new society, while 45.8% experienced it intermittently, and 39.8% never felt isolated (*M* = 1.75, SD = 0.692). The lower mean score and smaller standard deviation for isolation compared to alienation suggest that while spatial displacement occurred, social networks were successfully reconstructed or maintained. This finding was corroborated by qualitative data. Key informant K3 explained that “*the Nubian social structure is deeply rooted in collectivism and solidarity. This has led Nubians to create strong social support networks, even in places of displacement, through social ties and community organizations, particularly in the diaspora.”* Another informant (K11) described specific mechanisms: “*Each family was keen to establish its own guesthouse, which served as a permanent social gathering place, preserving the customs of meeting and bonding among individuals. This type of initiative served as a social safety valve that alleviated feelings of isolation.”*

From a Social Identity Theory perspective, these patterns reflect differential identity threat responses. The 34.6% experiencing persistent alienation demonstrate what Tajfel and Turner (2004) describe as identity discontinuity when the social environment no longer validates group membership, creating dissonance between self-categorization and contextual feedback. Conversely, 44.2% who never felt alienated may represent individuals for whom Nubian identity was less central to self-concept, facilitating adaptation. The successful mitigation of social isolation through cultural practices (guesthouses, community organizations) exemplifies how in-group cohesion serves as a buffer against environmental threats to identity. These boundary-maintaining practices sustained group distinctiveness and psychological belonging despite hostile or indifferent out-group contexts.

#### 5.3.2 Nostalgia as distress and cultural resistance

The nostalgic dimension of displacement revealed a complex emotional terrain. When asked about sadness upon remembering pre-displacement life, responses distributed across all three categories evenly: 35.9% never experienced this sadness, 46.6% experienced it sometimes, and 17.5% felt it constantly (*M* = 2.11, SD = 0.903). However, when asked specifically whether memories of the Nubian village increased sadness in exile, the distribution shifted slightly, with 34.6% never affected, 44.2% sometimes affected, and 21.2% always affected. Most strikingly, 46.6% believed their psychological comfort would never return to pre-displacement levels, with only 17.8% experiencing this belief intermittently and 35.6% not holding this belief. The statistical closeness of mean values across these items (2.10–2.11) indicates high interdependence between nostalgic memory and perceived psychological damage.

The qualitative data provided generational and experiential depth to these patterns. Informant K12 emphasized the elderly’s particularly acute suffering: *“The psychological experience of displacement for Nubians was bitter, especially when it involved the loss of home, heritage, and deep community ties. The elderly experienced a profound sense of loss and uprooting from the land to which they were emotionally and culturally attached. This created a lasting sense of longing and internal pain, making it extremely difficult to adapt to the new reality. Some have continued to talk about their old Nubian past to this day*.” The persistence of memory work that continues to talk about their old Nubian past to this day suggests that for some, nostalgia functions not as adaptation but as resistance to assimilation.

This resistance took cultural forms. Informant K15 explained: “There are still adults who harbor deep nostalgia for the old days, a sentiment evident even in the songs and folk art that spread after the displacement. It was a means of expressing longing and rejection of the new reality, a constant attempt to cement memories in the psyche.”

The transformation of nostalgia into artistic expression, such as songs and folk art, represents what Social Identity Theory terms “social creativity”: when direct social mobility or changing objective conditions is impossible, groups redefine the dimensions on which they compare themselves to out-groups. By elevating cultural heritage and memory as valued dimensions, Nubians maintained positive group evaluation despite material disadvantage. This cultural resistance through nostalgia served dual functions: processing grief while simultaneously asserting collective identity against assimilative pressures.

The finding that approximately one-third remained unaffected by nostalgic distress, while another third experienced it constantly, underscores the heterogeneity in psychological responses. This variability likely reflects differences in age (elders more affected), degree of pre-displacement attachment to place, and individual differences in identity centrality. Those for whom place-based Nubian identity was core to self-concept would experience decontextualization more traumatically than those with more portable or less territorialized identities.

### 5.4 Identity formation and reconstruction following displacement

The examination of identity processes revealed three interrelated dynamics: emotional responses to identity threats, the conditional nature of identity acceptance in new environments, and the apparent paradox of simultaneous identity loss and preservation efforts. These patterns are consistent with empirical evidence showing that threats to valued social identities can elicit negative affective responses, particularly when group membership is central to the self-concept, and may simultaneously strengthen identification with the threatened group (McCoy and Major, 2003; Fath and Proudfoot, 2024). Table 4 presents the distribution of responses across five identity-related items.

**Table 4:** Cultural identity perceptions and preservation efforts (*N* = 382).

Item	Never	Sometimes	Always	M	SD
	*f* (%)	*f* (%)	*f* (%)		
Identity not accepted in the new environment	146 (38.2)	184 (48.2)	52 (13.6)	1.75	0.68
Tense when downplaying identity	150 (39.3)	52 (13.6)	180 (47.1)	2.08	0.93
Displacement caused identity loss	59 (15.4)	140 (36.6)	183 (47.9)	2.32	0.73
Preserve the Nubian language	148 (38.7)	50 (13.1)	184 (48.2)	2.09	0.93
Angry when heritage neglected	151 (39.5)	51 (13.4)	180 (47.1)	2.08	0.93

#### 5.4.1 Emotional polarization in response to identity threat

The most striking pattern in Table 3 is the precise alignment of emotional responses to identity threat. Exactly 47.1% reported feeling tense when required to downplay aspects of their Nubian cultural identity, and identically 47.1% expressed anger when others neglected their heritage. This numerical coincidence, supported by identical mean scores (*M* = 2.08) and nearly matching standard deviations (SD = 0.927 and 0.929), points to a shared cognitive-emotional pathway. When identity is threatened either through forced concealment or external devaluation, approximately half the population responds with intense negative emotions (tension, anger). The consistency across these distinct manifestations of threat suggests that for high identifiers, any form of identity invalidation triggers similar affective responses.

However, this represents only half the story. Approximately 39% reported never experiencing tension from identity concealment or anger at heritage neglect, indicating substantial heterogeneity in identity salience. Among these polarized groups, only 13–14% reported intermediate or occasional emotional responses. This tripartite distribution with a weak middle ground suggests that identity centrality functions somewhat categorically rather than continuously: Nubian identity is either core to self-concept (eliciting strong emotional responses to threat) or peripheral (eliciting minimal response), with relatively few individuals occupying middle positions.

Qualitative data contextualized this polarization. Informant K30 explained: *“The psychological effects of displacement are diverse, but they often manifest as a sense of loss. The erosion of heritage and community ties leads to states of anxiety, depression, and even a feeling of emptiness. The threat to one’s very identity creates a strong psychological response, sometimes anger, sometimes denial or withdrawal.*”

This narrative captures the range of possible reactions while acknowledging that intensity varies based on identity centrality. From Social Identity Theory, these patterns reflect that group membership becomes especially salient under perceived threat. For individuals whose self-concept is strongly anchored in Nubian identity, threats to that identity constitute threats to the self, necessitating defensive emotional and behavioral responses to protect self-esteem. For low identifiers, the same objective conditions (pressure to downplay identity, heritage neglect) pose minimal psychological threat because the threatened identity is not central to self-definition. The qualitative findings confirmed that this identity threat produces a spectrum of psychological responses, including anxiety, depression, a feeling of emptiness, anger, denial, and withdrawal, with the intensity of the response directly proportional to the centrality of Nubian identity to the individual’s self-concept.

#### 5.4.2 Conditional acceptance and identity indeterminacy

The largest proportion of respondents (48.2%) reported that their Nubian identity was sometimes accepted in the new environment, while 38.2% felt it was accepted, and 13.6% experienced consistent rejection. This distribution reflects what can be termed “identity indeterminacy” belonging that is context-dependent, contingent, and unstable. The moderate mean (*M* = 1.75) and relatively low standard deviation (SD = 0.677) indicate consensus around conditional rather than absolute acceptance or rejection.

Qualitative data illuminated the mechanisms and consequences of this conditionality. Informant K25 observed: “*Relocation led to changes in the cultural and social environment surrounding the Nubians, and this made the adaptation process vary from one person to another. The older generations were closer to the land and traditions, making it difficult for them to integrate, but even the younger generations, despite their flexibility, faced challenges in asserting their identity in environments that may not recognize or understand their culture*.”

This generational analysis suggests that while younger Nubians may possess greater adaptive capacity, structural barriers, lack of recognition, and cultural misunderstanding constrain even their integration. From Social Identity Theory, conditional acceptance reflects boundary permeability dynamics. When group boundaries are permeable, individual mobility becomes theoretically possible but practically contingent. Nubians face selective validation: their identity may be accepted in certain contexts (within the Nubian community and at cultural festivals) while rejected or ignored in others (workplaces, bureaucratic settings, and interactions with non-Nubian neighbors). This creates psychological strain as individuals must continuously navigate when and how to assert or conceal group membership. For the 13.6% experiencing consistent rejection, boundaries are effectively impermeable; no individual mobility option exists, only a stigmatized minority status. This group likely experiences the most severe psychological distress, though the small sample size (*n* = 52) limits demographic analysis of this particularly vulnerable subgroup. The qualitative findings clarified that this conditionality is strongly shaped by generational position: older generations, more deeply rooted in the land and traditions, face greater difficulty integrating, while younger generations, despite greater flexibility, still encounter structural barriers and cultural misrecognition in asserting their Nubian identity.

#### 5.4.3 The paradox of loss and preservation: identity compensation

The most theoretically intriguing finding concerns the simultaneous experience of identity loss and preservation efforts. Nearly half the sample (47.9%) reported a perceived loss of part of their identity following displacement, the highest mean score among all identity items (*M* = 2.32, SD = 0.728). Yet an almost identical proportion (48.2%) reported actively preserving the Nubian language as part of their identity (*M* = 2.09, SD = 0.929). While these percentages appear similar, the mean difference (0.23 points) and divergent standard deviations suggest these are not equivalent phenomena but rather represent a compensatory dynamic.

The lower standard deviation for perceived identity loss (0.728) compared to language preservation (0.929) indicates that loss is experienced relatively informally; most respondents recognize some erosion of identity, even if the degree varies. In contrast, the higher variability in language preservation reflects divergent responses: some intensively preserve language (scoring 3), others moderately (scoring 2), and still others do not engage in preservation (scoring 1). Importantly, only 15.4% reported no sense of identity loss, but 38.7% reported not preserving language. This asymmetry indicates that experiencing loss does not automatically trigger preservation efforts; individual agency, resources, and commitment mediate this relationship.

Qualitative accounts captured both the loss and the compensatory response. Respondent R5 articulated: “After the displacement, I truly feel like I’ve lost a large part of my identity, not only because of the distance from my land, but also because of the distance from the customs and traditions we lived by every day. Nevertheless, I’m trying my best to make up for this by teaching my children the Nubian language and ensuring we use it at home, as a bridge connecting us to our roots.”

The phrase “make up for this” explicitly frames language preservation as compensation for loss. The language becomes a portable cultural marker that can be maintained despite spatial displacement, a symbolic anchor when territorial anchors have been severed.

Key informant K14 situated language preservation within broader cultural resistance: “Preserving heritage was a natural response to what they had lost. Nubians used music, dance, language, and even building styles to affirm their identity, not just express it. This was a form of cultural resistance that emerged after displacement, as if every daily activity was an affirmation: “We are still here.”

The framing of preservation as resistance, “We are still here”, aligns with Social Identity Theory’s concept of identity-protective behavior under threat. When group identity is devalued or threatened with extinction, members engage in strategies to restore positive distinctiveness and maintain group continuity. Language preservation serves multiple functions: it differentiates the in-group from out-groups, transmits cultural knowledge to younger generations, and provides a daily enactment of group membership that reinforces psychological belonging. The qualitative findings confirmed that language preservation is a conscious compensatory act. Nubians teach their children the language at home as a bridge to their roots, making up for the distance from the land. More broadly, heritage preservation through music, dance, and language was described as a form of cultural resistance: a daily affirmation that “we are still here.”

### 5.5 Multivariate patterns: demographic differences and psychological profiles

To examine how psychological patterns varied across demographic groups and to identify distinct psychological profiles within the sample, multivariate analyses were conducted. MANOVA examined demographic differences while cluster analysis identified respondent subgroups.

#### 5.5.1 Education and marital status as sources of variation

Multivariate Analysis of Variance testing demographic variables as predictors of the four psychological outcome scales (Alienation & Grief, Cultural Identity, Social Support Loss, Psychological Belonging) revealed that only education level and marital status produced significant multivariate effects. Education level showed a significant multivariate effect [Wilks’ Λ = 0.897, *F*(16, 1,143) = 2.59, *p* = 0.001], as did marital status [Wilks’ Λ = 0.919, *F*(12, 992) = 2.69, *p* = 0.001]. In contrast, age group (Wilks’ Λ = 0.966, *p* = 0.374), gender (Wilks’ Λ = 0.990, *p* = 0.445), and family size (Wilks’ Λ = 0.964, *p* = 0.841) showed no significant multivariate effects.

The education findings challenge assumptions that higher education serves as a protective factor against displacement-related distress. Examination of group means revealed that illiterate respondents reported the highest Psychological Belonging (*M* = 9.42) and lowest Social Support Loss (*M* = 8.25), while respondents with above-intermediate education exhibited the highest Alienation scores (*M* = 13.48). This pattern suggests that formal education may actually heighten awareness of cultural loss and articulation of identity threats. Those with more education possess greater cognitive and linguistic resources to conceptualize and express alienation, while less educated respondents may experience belonging through more concrete, relational mechanisms less dependent on abstract identity concepts. Alternatively, educational attainment may correlate with urbanization, exposure to cultural assimilation pressures, or employment in contexts requiring identity negotiation, all of which could increase alienation.

Post-hoc comparisons using Tukey’s HSD test revealed that the difference in Alienation scores between above-intermediate education (*M* = 13.48) and illiterate respondents (*M* = 10.25) was significant (*p* = 0.012), while differences between other education levels were not statistically significant. This suggests a threshold effect wherein education beyond the secondary level specifically heightens alienation awareness.

Marital status differences were equally informative. Widowed respondents showed the highest Alienation (*M* = 13.14), Cultural Identity (*M* = 11.33), and Psychological Belonging (*M* = 9.43), suggesting that spousal loss compounds displacement trauma, intensifying both identity salience and distress. The bereavement may trigger heightened attachment to other sources of identity and belonging as compensation for personal loss. Conversely, divorced respondents exhibited the lowest Cultural Identity scores (*M* = 8.67), indicating that marital dissolution may be associated with weakened cultural connections, possibly due to disrupted family-based identity transmission or social marginalization within the community. The fact that age, gender, and family size showed no significant effects indicates that displacement is experienced as a collective community trauma that transcends these demographic boundaries, affecting all subgroups similarly regardless of these individual characteristics. Tables 5–7 present the full MANOVA results and scale means by education level and marital status.

**Table 5:** MANOVA results by demographic variable (*N* = 382).

Demographic variable	Wilks’ Λ	F-statistic (df)	*p*-value	Decision
Education level	0.897	*F*(16, 1,143) = 2.59	0.001	Significant **
Marital status	0.919	*F*(12, 992) = 2.69	0.001	Significant **
Age group	0.966	1.08	0.374	Not Significant
Gender	0.990	0.93	0.445	Not Significant
Family size	0.964	0.69	0.841	Not Significant

Dependent variables: Alienation & Grief, Cultural Identity, Social Support Loss, Psychological Belonging. **p < 0.01.

**Table 6:** Psychological scale means by education level.

Education level	Alienation and grief	Cultural identity	Social support loss	Psych. belonging
Illiterate	10.25	10.83	8.25	9.42
Intermediate	12.82	10.43	9.16	8.62
Above intermediate	13.48	10.90	9.57	8.07
University graduate	11.77	10.07	9.12	8.48
Post-studies	12.10	10.62	9.33	8.50

Above-intermediate education: highest Alienation (*M* = 13.48). Illiterate: highest Psychological Belonging (*M* = 9.42).

**Table 7:** Psychological scale means by marital status.

Marital status	Alienation and grief	Cultural identity	Social support loss	Psych. belonging
Single	11.49	10.51	9.33	8.39
Married	12.52	10.31	9.11	8.50
Divorced	11.86	8.67	9.57	8.10
Widowed	13.14	11.33	8.81	9.43

Widowed: highest Alienation (*M* = 13.14) and Cultural Identity (*M* = 11.33). Divorced: lowest Cultural Identity (*M* = 8.67).

#### 5.5.2 Four distinct psychological adaptation profiles

K-means cluster analysis partitioned the sample into four groups based on their psychological scale profiles, revealing heterogeneous adaptation patterns. Table 4 presents the cluster characteristics.

Cluster 1 (*n* = 88, 23.0%) showed low Cultural Identity (*M* = 7.55) combined with moderate Alienation (*M* = 11.00), suggesting that individuals have partially detached from their Nubian cultural roots, possibly as an adaptive strategy. This profile may represent successful assimilation or, alternatively, identity loss without compensatory preservation (Table 8).

**Table 8:** Cluster profiles of psychological adaptation (*N* = 382).

Cluster	*n* (%)	Alienation and grief	Cultural identity	Psychological belonging	Social support loss	Profile description
1	88 (23.0)	11.00	7.55	8.16	7.36	Low identity/moderate distress
2	92 (24.1)	9.35	10.71	8.53	7.45	Well-adjusted/moderate identity
3	115 (30.1)	15.33	11.87	9.78	7.80	High distress/strong identity
4	87 (22.8)	12.54	10.25	7.43	7.39	Moderate distress/weak belonging

Cluster 2 (*n* = 92, 24.1%) exhibited the lowest Alienation (*M* = 9.35) alongside a moderate level of Cultural Identity (*M* = 10.71), representing the best-adjusted group of individuals who maintain cultural connections while experiencing minimal displacement-related distress. This may reflect successful bicultural identity integration or residence in more supportive environments.

Cluster 3 (*n* = 115, 30.1%) was the largest group and, theoretically, the most significant. These individuals showed the highest scores on Alienation (*M* = 15.33), Cultural Identity (*M* = 11.87), and Psychological Belonging (*M* = 9.78), reflecting the coexistence of high alienation, strong cultural identity, and robust psychological belonging. These respondents maintain strong cultural connections and experience robust belonging to the Nubian community, yet simultaneously report high levels of alienation and grief. This profile demonstrates that cultural preservation and psychological suffering are not mutually exclusive but may coexist among those with a strong attachment to their heritage and a profound sense of loss associated with displacement.

Finally, Cluster 4 (*n* = 87, 22.8%) demonstrated moderate Alienation (*M* = 12.54) but the lowest Psychological Belonging (*M* = 7.43), indicating individuals who struggle with place attachment and community connection despite moderate cultural engagement. This disconnection despite cultural involvement suggests that belonging is not solely determined by identity maintenance but also requires environmental validation and social inclusion.

The existence of four distinct profiles, rather than a single normative response, underscores the limitations of universal intervention approaches. Each cluster requires different support: Cluster 1 may benefit from cultural reconnection programs; Cluster 2 represents resilience to be leveraged; Cluster 3 requires trauma-informed mental health services that validate cultural identity; and Cluster 4 needs social integration support focused on building belonging.

### 5.6 Statistical associations, mediation, and moderation

Regression, mediation, and moderation analyses were conducted to examine statistical associations among the psychological constructs. Multiple regression examining Psychological Belonging in relation to the three other psychological variables (Alienation & Grief, Cultural Identity, Social Support Loss) revealed that the model explained 10.4% of variance (*R*^2^ = 0.104, Adjusted *R*^2^ = 0.097). Alienation showed the strongest association with Psychological Belonging (β = 0.250, *p* < 0.001, accounting for 57.05% of relative importance), followed by Cultural Identity (β = 0.150, *p* = 0.003, 34.06% relative importance). Social Support Loss was not significantly associated with Belonging in the regression model (β = −0.039, *p* = 0.427), indicating no statistically significant independent association with Psychological Belonging.

The positive coefficient for Alienation indicates a positive association between Alienation and Psychological Belonging. From a Social Identity Theory perspective, individuals who experience alienation from the out-group (mainstream Egyptian society) may intensify their attachment to the in-group (Nubian community) under conditions of perceived exclusion or identity threat. In-group identification may therefore provide a source of validation and positive distinctiveness when out-group acceptance is perceived as limited. This pattern was most pronounced in Cluster 3, where high alienation coexisted with high belonging.

Mediation analysis examined potential indirect associations among the study variables. Three models were examined: (1) Cultural Identity → Social Support Loss → Belonging, (2) Alienation → Social Support Loss → Belonging, and (3) Cultural Identity → Alienation → Belonging. Only Model 3 showed a statistically significant indirect effect (Sobel Z = 3.24, *p* = 0.001), with Alienation accounting for 26.5% of the statistical association between Cultural Identity and Belonging. Specifically, Cultural Identity was positively associated with Alienation (path a: β = 0.249, *p* < 0.001), which was in turn positively associated with Belonging (path b: β = 0.165, *p* < 0.001), while Cultural Identity retained a significant direct association with Belonging (c’: β = 0.114, *p* = 0.003).

Moderation analysis examined whether relationships varied across levels of other variables. Cultural Identity significantly moderated the Alienation-Belonging relationship (β_interaction = −0.143, *p* = 0.028, Δ*R*^2^ = 0.012). Simple slopes analysis revealed that the positive relationship between Alienation and Belonging was strongest for individuals with low Cultural Identity (slope = 0.396) and weakest for those with high Cultural Identity (slope = 0.110). This pattern is consistent with a potential buffering role of Cultural Identity, as the association between Alienation and Belonging was weaker among respondents with stronger Cultural Identity. Conversely, among respondents with lower Cultural Identity, the positive association between Alienation and Belonging was stronger. This moderation pattern highlights Cultural Identity as a potentially relevant consideration in psychosocial interventions addressing alienation and belonging. Tables 9–11 summarize the mediation, moderation, and simple slopes analyses for the Alienation-Belonging relationship.

**Table 9:** Mediation analysis (Sobel test)—indirect associations with psychological belonging (*N* = 382).

Pathway (Model)	Indirect effect	Sobel Z	*p*-value	Result
Model 1: cultural identity → social support loss → belonging	−0.001	−0.240	0.811	No mediation
Model 2: alienation → social support loss → belonging	0.003	0.648	0.517	No mediation
Model 3: cultural identity → alienation → belonging	0.041	3.239	0.001	Partial mediation (26.5%) **

**p < 0.01. Model 3: path a (β = 0.249, *p* < 0.001); path b (β = 0.165, *p* < 0.001); direct effect c’ (β = 0.114, *p* = 0.003).

**Table 10:** Moderation analysis—cultural identity moderating alienation → belonging (*N* = 382).

Interaction term	*R* ^2^	Δ*R*^2^	β (Interaction)	*p*-value	Decision
Alienation × cultural identity → belonging	0.114	0.012	−0.143	0.028	Significant*

*p < 0.05. Cultural Identity buffers Alienation→Belonging: strongest at low identity (slope = 0.396), weakest at high identity (slope = 0.110).

**Table 11:** Simple slopes—association between alienation and belonging at different levels of cultural identity.

Cultural identity level	Simple slope	Interpretation
Low identity (−1 SD)	0.396	Strong positive association at low Cultural Identity
Mean identity	0.253	Moderate positive effect
High identity (+1 SD)	0.110	Weak: cultural identity independently anchors belonging

*p < 0.05 for interaction term (β = −0.143, *p* = 0.028).

## 6 Discussion

The findings confirm that Nubian displacement represents profound identity disruption, consistent with Social Identity Theory’s emphasis on group-based self-concept. The four distinct psychological factors, grief, displacement-related alienation, cultural heritage concern, and adaptation/isolation, validate the multidimensional nature of identity processes under threat.

The 34.6% experiencing persistent alienation and 44.2% reporting lost inner security reflect what Tajfel and Turner (2004) describe as identity discontinuity resulting from weakened in-group recognition in new environments. From the SIT perspective, alienation arises when the social context no longer validates the “Nubian” identity prototype, creating dissonance between self-categorization and environmental feedback. This aligns with contemporary SIT work treating identity as dynamic and context-dependent (Curşeu et al., 2020).

However, the notable 44.2% who never experienced alienation reveal heterogeneity in identity processes. This may reflect individual differences in identity centrality for some, Nubian identity is peripheral to self-concept, allowing easier adaptation; for others, it is core, making displacement more traumatic. The cluster analysis confirmed this heterogeneity, identifying four distinct profiles ranging from low-identity/moderate-distress to high-identity/high-distress.

The relatively lower social isolation (14.4% constant, 45.8% intermittent) compared to alienation suggests successful in-group cohesion maintenance despite displacement. Qualitative data revealed mechanisms: traditional guesthouses, community associations, and family networks functioned as identity-preserving spaces. From SIT, these represent boundary-maintaining practices that sustain group distinctiveness and provide psychological belonging even in indifferent out-group contexts (Bouncken et al., 2022). The qualitative findings reinforced this pattern, showing that the Nubian social structure, rooted in collectivism and solidarity, enabled the community to rebuild social support networks through communal spaces such as the guesthouse, which served as a permanent social gathering place and a social safety valve that alleviated feelings of isolation.

The finding that 35.9–46.6% experience persistent nostalgia-related distress, while others use nostalgia for cultural resistance (art, music, storytelling), exemplifies SIT’s social comparison process. When displaced groups perceive negative distinctiveness (current situation compares unfavorably to the past), two responses emerge: (1) psychological withdrawal and grief, or (2) social creativity redefining dimensions of comparison to restore positive distinctiveness (Kanzola et al., 2024).

The transformation of nostalgia into cultural expression represents the latter strategy. By elevating cultural heritage as the valued dimension rather than material conditions, Nubians maintain a positive group evaluation despite the hardships of displacement. This aligns with Gallamaso (2024) describing nostalgia’s dual functions, comfort and distress, and supports Xi and Hwang (2011) documenting memory work as identity reconstruction. The qualitative findings added further depth, revealing that this nostalgia was felt most acutely by the elderly as an existential wound tied to the loss of home, heritage, and the Nile. Moreover, it manifested collectively through songs and folk art that spread after displacement as a means of expressing longing, rejecting the new reality, and cementing collective memory across generations.

The apparent contradiction, 47.9% reporting identity loss while 48.2% actively preserving language, reflects not a paradox but compensatory identity work. From SIT, when identity is threatened, members engage in protective behaviors to restore self-esteem and group coherence (Chen et al., 2024). Language preservation functions as identity-protective behavior, attempting to maintain symbolic group boundaries when territorial boundaries have been violated.

The 47.1% experiencing stress when concealing identity, alongside the identical 47.1% expressing anger at heritage neglect, demonstrates an identity threat response. When out-group members devalue in-group attributes, members who strongly identify experience this as a personal threat, triggering defensive emotions (Abrams, 2001). The polarization (39.5% experiencing no anger) reflects SIT’s distinction between high and low identifiers, only those for whom Nubian identity is a central experience, and devaluation as threatening.

The most striking finding is the positive relationship between Alienation and Belonging (*r* = 0.286, β = 0.250), with Alienation mediating 26.5% of the Identity→Belonging pathway. This counterintuitive pattern makes sense through SIT: heightened in-group identification under threat.

When individuals experience alienation from the out-group (mainstream society), social comparison processes may intensify in-group attachment as a compensatory mechanism. Alienation signals that positive distinctiveness cannot be achieved through out-group acceptance, redirecting individuals toward in-group sources of self-esteem. The Cluster 3 profile (30% of the sample), high alienation, high identity, and high belonging, embodies this dynamic.

The significant indirect association involving Cultural Identity, Alienation, and Belonging is consistent with an SIT interpretation in which stronger cultural identification may coincide with greater awareness of cultural distance from the wider social environment and stronger in-group attachment. This interpretation also aligns with SIT’s emphasis on depersonalization under strong group salience, whereby self-perception may become more closely aligned with the group prototype, reinforcing collective belonging (Hogg, 2016).

The moderation analysis showed that the positive Alienation–Belonging association was weaker at higher levels of Cultural Identity and stronger at lower levels. This pattern is consistent with the possibility that established cultural identification may be associated with more stable belonging.

The significant MANOVA effects for education and marital status, but not age, gender, or family size, suggest that identity salience varies more by social than demographic factors. The absence of significant age-related differences should be interpreted in light of the generational composition of the sample. Because most respondents were born after the 1963–1964 relocation, the reported psychological and identity-related experiences are understood to reflect post-resettlement lived experience, together with intergenerational and collective memory processes, rather than direct displacement experience alone. Higher education’s association with increased alienation (*M* = 13.48) may reflect greater awareness of cultural loss, consistent with research showing education enhances critical consciousness.

Widowed respondents’ profile (highest alienation, identity, and belonging) suggests compound loss, spousal death plus displacement, creates a dual identity threat, intensifying in-group attachment. Divorced respondents’ lowest identity (*M* = 8.67) indicates that marital dissolution undermines family-based identity transmission.

The findings align with displacement scholarship, emphasizing multidimensional impacts (Bose, 2018; Cheng, 2022) while extending understanding through the SIT lens. The heterogeneous responses (four cluster profiles) resonate with McGrath et al. (2022)documenting divergent coping strategies and Walker et al. (2023) revealing polarized displacement experiences.

The gender role changes and occupational transformation (only 1.3% in agriculture) reflect development-induced displacement patterns documented by Rajan (2018) and Priyadarshini and Dulla (2023). The successful social network reconstruction despite spatial displacement supports Lakshman and Azam (2023), emphasizing close-knit group maintenance, while the transformation of nostalgia into cultural expression illustrates Hay et al. (2019), observing how nostalgia complicates integration in culturally incongruent environments.

### 6.1 Theoretical contributions and practical implications

This study advances displacement scholarship and extends Social Identity Theory by showing that post-displacement psychological outcomes are shaped by social categorization, comparison, and in-group identification. First, the findings support the relevance of Social Identity Theory in displacement settings by demonstrating that identity processes explain variation in alienation, inner security, and belonging. Second, alienation from the dominant environment was positively associated with in-group belonging. Third, Cultural Identity showed both a significant indirect association with Belonging through Alienation and a significant moderating role in the Alienation–Belonging relationship. Third, cultural identity functions as both a mediator and a moderator. It mediates the pathway from identity to belonging through alienation and moderates the alienation-belonging association by buffering disruption at higher identity levels. Fourth, four adaptation profiles demonstrate heterogeneous displacement trajectories, challenging monolithic interpretations. Fifth, language preservation reflects a compensatory mechanism of identity maintenance and active agency in identity reconstruction under threat.

These findings also inform psychosocial programming for displaced populations. The distinct profiles indicate that uniform interventions are insufficient and support should be differentiated. Cluster 3 requires trauma-informed services that address distress while legitimizing cultural attachment. Cluster 4 requires integration-focused support targeting social inclusion and belonging. Cluster 1 benefits from cultural reconnection that rebuilds identity resources, while Cluster 2 can be strengthened through community participation and leadership that mobilizes resilience. Finally, the protective role of cultural identity suggests that language revitalization, heritage education, and culturally grounded community programming should be framed as psychosocial interventions with mental health value rather than as separate cultural activities.

## 7 Conclusion

This mixed-methods study examined the long-term psychological and identity consequences of forced displacement on the Nubian community resettled in Kom Ombo, more than six decades after the construction of the Aswan High Dam. Grounded in Social Identity Theory and integrating quantitative survey data from 382 participants with qualitative interviews from 60 participants, the study addressed two interconnected research questions: what are the psychological impacts of forced displacement on the sense of belonging among resettled Nubians, and what psychological and identity patterns does displacement reveal, and how does cultural identity function as a mediator and moderator in the relationship between alienation and belonging.

The findings indicate substantial heterogeneity in psychological responses within the resettled Nubian community. Four empirically distinct adaptation profiles were identified: a culturally detached group managing through partial disengagement; a well-adjusted group maintaining a moderate level of cultural identity with low distress; a high-distress group simultaneously exhibiting a strong cultural identity and robust community belonging; and a disconnected group struggling with belonging despite cultural engagement. The most theoretically significant finding is that 30% of participants, the largest cluster, combined high alienation with strong identity and strong belonging, challenging linear assumptions about the relationship between cultural connectedness and distress.

A counterintuitive positive association between alienation and belonging emerged as a central finding: higher alienation from mainstream Egyptian society was associated with stronger belonging to the Nubian community. Mediation analysis identified a significant indirect association between Cultural Identity and Belonging through Alienation, with Alienation accounting for 26.5% of the statistical association (Sobel Z = 3.24, *p* = 0.001). Moderation analysis further showed that the Alienation–Belonging association varied significantly across levels of Cultural Identity (β = −0.143, *p* = 0.028), with the association weaker at higher levels of Cultural Identity. These findings are consistent with an SIT interpretation of intensified in-group identification under conditions of perceived cultural threat.

Demographic patterns revealed that education level and marital status, rather than age, gender, or family size, significantly differentiated psychological outcomes, suggesting that displacement functions as a collective community trauma that transcends basic demographic boundaries. Higher education was associated with heightened alienation, while widowed participants showed the most complex psychological profiles, combining the grief of bereavement with the grief of displacement.

Recommendations: Interventions must be differentiated by psychological profile rather than applied uniformly. The high-distress group requires trauma-informed services that validate rather than pathologize cultural attachment. The disconnected group needs integration-focused support addressing social belonging. The detached group would benefit from cultural reconnection programs that rebuild identity resources. Across all groups, language revitalization, heritage education, and culturally grounded community programming should be recognized as psychosocial interventions with direct mental health value, not peripheral cultural activities.

Limitations: The cross-sectional design precludes causal inference and longitudinal tracking of identity trajectories. Possible sampling bias toward more educated participants may limit generalizability to the broader Nubian population. Reliance on self-report measures and a three-point response scale restricts the depth of psychological measurement. Future research should pursue longitudinal designs to track intergenerational identity change, comparative studies in other dam-displaced communities, and controlled trials to test the efficacy of culturally grounded psychosocial interventions.

## Data Availability

The original contributions presented in the study are included in the article/supplementary material, further inquiries can be directed to the corresponding author.
